# Low concentrations of medium-sized HDL particles predict incident CVD in chronic kidney disease patients

**DOI:** 10.1016/j.jlr.2023.100381

**Published:** 2023-04-24

**Authors:** Baohai Shao, Farsad Afshinnia, Anna V. Mathew, Graziella E. Ronsein, Carissa Thornock, Angela D. Irwin, Mayank Kansal, Panduranga S. Rao, Mirela Dobre, Sadeer Al-Kindi, Matthew R. Weir, Alan Go, Jiang He, Jing Chen, Harold Feldman, Karin E. Bornfeldt, Subramaniam Pennathur, Michigan Kidney Translational Core CPROBE Investigator Group and the CRIC Study Investigators, Matthias Kretzler, Debbie Gipson, Markus Bitzer, Crystal Gadegbeku, Keith Bellovich, Zeenat Bhat, Susan Massengill, Kalyani Perumal, Lawrence J. Appel, Debbie L. Cohen, James P. Lash, Robert G. Nelson, Mahboob Rahman, Vallabh O. Shah, Mark L. Unruh

**Affiliations:** 1 Department of Medicine, UW Medicine Diabetes Institute, University of Washington, Seattle, WA, USA; 2 Division of Nephrology, Department of Internal Medicine, University of Michigan, Ann Arbor, Michigan, USA; 3 Department of Cardiology, University of Illinois at Chicago, Chicago, IL, USA; 4 Division of Nephrology and Hypertension, Case Western Reserve University, Cleveland, OH, USA; 5 Division of Nephrology, Department of Medicine, University of Maryland School of Medicine, Baltimore, MD, USA; 6 Department of Health System Science, Kaiser Permanente Bernard J. Tyson School of Medicine, Pasadena, CA, USA; 7 Department of Epidemiology, Tulane University School of Public Health and Tropical Medicine, New Orleans, LA, USA; 8 Department of Medicine, Tulane University School of Medicine, New Orleans, LA, USA; 9 Department of Biostatistics, Epidemiology and Informatics, Perelman School of Medicine, University of Pennsylvania, Philadelphia, PA, USA; 10 Department of Molecular and Integrative Physiology, University of Michigan, Ann Arbor, MI, USA

**Keywords:** cardiovascular disease, case-control study, cholesterol efflux capacity, CKD, high density lipoprotein, HDL-C levels, HDL particle concentration, incident CVD, ion mobility analysis, medium HDL-P

## Abstract

Patients with chronic kidney disease (CKD) are at high risk for CVD. However, traditional CVD risk factors cannot completely explain the increased risk. Altered HDL proteome is linked with incident CVD in CKD patients, but it is unclear whether other HDL metrics are associated with incident CVD in this population. In the current study, we analyzed samples from two independent prospective case-control cohorts of CKD patients, the Clinical Phenotyping and Resource Biobank Core (CPROBE) and the Chronic Renal Insufficiency Cohort (CRIC). We measured HDL particle sizes and concentrations (HDL-P) by calibrated ion mobility analysis and HDL cholesterol efflux capacity (CEC) by cAMP-stimulated J774 macrophages in 92 subjects from the CPROBE cohort (46 CVD and 46 controls) and in 91 subjects from the CRIC cohort (34 CVD and 57 controls). We tested associations of HDL metrics with incident CVD using logistic regression analysis. No significant associations were found for HDL-C or HDL-CEC in either cohort. Total HDL-P was only negatively associated with incident CVD in the CRIC cohort in unadjusted analysis. Among the six sized HDL subspecies, only medium-sized HDL-P was significantly and negatively associated with incident CVD in both cohorts after adjusting for clinical confounders and lipid risk factors with odds ratios (per 1-SD) of 0.45 (0.22–0.93, *P* = 0.032) and 0.42 (0.20–0.87, *P* = 0.019) for CPROBE and CRIC cohorts, respectively. Our observations indicate that medium-sized HDL-P—but not other-sized HDL-P or total HDL-P, HDL-C, or HDL-CEC—may be a prognostic cardiovascular risk marker in CKD.

Patients with chronic kidney disease (CKD) exhibit an elevated CVD risk in all stages of the disease compared with the general population, and CVD is the primary cause of morbidity and mortality in patients with CKD (1). However, traditional cardiovascular risk factors do not explain the high incidence of CVD frequently seen in this population. One important factor could be altered levels of HDL as low HDL-C is a common feature in patients with CKD (2). HDL is a circulating, noncovalent assembly of amphipathic proteins and lipids. Clinical and epidemiological studies show a robust, negative association of HDL-C levels with CVD risk in the general population (3). However, several lines of evidence suggest that genetically low concentrations of HDL-C do not causally associate with a high risk of CVD (4, 5). Moreover, elevating HDL-C in randomized clinical trials has not reduced CVD risk (6), suggesting that this metric does not reflect HDL’s proposed cardioprotective effects. It is therefore critical to identify new HDL metrics that capture HDL’s proposed cardioprotective effects. Several distinct pathways have been implicated in HDL’s cardioprotective effects. Animal studies provide strong evidence that one key cardioprotective effect of HDL is the promotion of cholesterol efflux from artery wall macrophages (7, 8). In the general population with normal kidney function, HDL’s ability to remove cholesterol from cultured macrophages strongly and negatively associates with CVD status (9). Importantly, the cholesterol efflux capacity (CEC) of serum HDL (HDL-CEC) is a stronger predictor of incident CVD than HDL-C (10, 11).

HDL in the circulation is a heterogeneous mixture of particles consisting of multiple subspecies with a diameter from 7 to 14 nm. The content of cholesterol in HDL subspecies can vary more than 4-fold. Thus, measurements of HDL-C levels do not necessarily indicate either the overall abundance of HDL particles or the distribution of the different subspecies. HDL particle concentration (HDL-P) and size might be better predictors of CVD risk than HDL-C (12). Multiple human studies demonstrated that diminished total HDL-P can be superior to reduced HDL-CEC or HDL-C levels in terms of cardiovascular risk prediction (12, 13). There are several techniques used to separate and measure HDL subpopulations (14). Most studies used NMR spectroscopy, in which the data of HDL-P is estimated by computational deconvolution from the differences in the spectral properties of the lipids carried by large, medium, and small HDL particles (15). Using this technique, levels of large HDL frequently display negative relationships with cardiovascular risk, whereas concentrations of small HDL particles typically reveal positive correlations with the risk (16, 17, 18, 19). In contrast to NMR, ion mobility analysis (IMA) can directly measure HDL particle numbers in large, medium, and small HDL, and comparable associations of small and large HDL particles with cardiovascular risk was revealed by this technique (20). Quantifying HDL-P with calibrated IMA (cIMA, HDL-P_IMA_) that we developed yields values in excellent agreement with our current understanding of the size distribution and stoichiometry of APOA1 per HDL particle, in contrast with other methods used to determine HDL-P (21).

Although low HDL-C levels are associated with CKD status (22), it is unclear whether HDL metrics associate with incident CVD events in CKD patients. In a previous study, we demonstrated that four HDL proteins, paraoxonase 1 (PON1), PON3, LCAT, and APOA4, are associated with future CVD events in patients at different stages of CKD (23). In the current study, we determined whether other HDL metrics are also associated with future CVD events in the patients of CKD. We used cIMA (21) to measure different-sized HDL-P subspecies including extra-small, small, medium, medium-large, large, and extra-large HDL-P. Our results demonstrated that, in both Clinical Phenotyping and Resource Biobank Core (CPROBE) and Chronic Renal Insufficiency Cohort (CRIC), two prospective studies on CVD risk in CKD patients, only medium HDL-P—but not other-sized HDL-P, total HDL-P, HDL-CEC, or HDL-C—significantly and negatively associated with incident CVD in subjects with CKD in models adjusted for clinical confounders and lipid risk factors.

## Materials and methods

### Subjects from two CKD cohorts

Established under the auspices of the George O’Brien Kidney Center at the University of Michigan, CPROBE is a multicenter cohort of 1,235 adult individuals with stages 1–5 CKD [according to the CKD Epidemiology Collaboration creatinine equation (CKD-EPI)] with high-quality biologic specimens and clinical data stored for future translational research. The Case-Control samples from the CPROBE cohort in the current study are nested in the parent CPROBE study. As of September 2017, out of the 1,235 adult participants aged 18 years or older in the parent CPROBE, 376 (30.4%) had cardiovascular outcomes. The selected samples in CPROBE were previously shown to be an unbiased representative of the entire CPROBE with participants’ characteristics and outcomes that do not differ with the unselected participants (24). We performed a prospective case-control study by analyzing stored plasma samples and compared 92 CPROBE subjects with and without incident CVD events (N = 46 each group). CVD outcome is defined as myocardial infarction (MI), angina, coronary artery bypass grafting or angioplasty/stenting of a coronary artery, stroke, peripheral arterial disease, congestive heart failure (CHF), or arrhythmia self-reported by patients and confirmed with electronic health record or by International Classification of Diseases-9 code review at CPROBE and non-CPROBE sites. We used plasma samples collected at the time of enrollment (between January of 2009 and July of 2015) and prospective clinical data available to us at the time of this study (follow-up 1–10 years). The 46 cases were individually matched to a control subject of the same sex and diabetes status in a one-to-one ratio. Both the CPROBE ancillary studies committee and the Institutional Review Board at the University of Michigan approved the analysis of these samples in a deidentified and blinded manner for HDL isolation, cholesterol efflux essay, and HDL particle analysis.

The CRIC study is an ongoing multicenter prospective study of cardiovascular and kidney outcomes in CKD patients. Detailed descriptions of the study are provided elsewhere (25). Briefly, the Phase-1 CRIC Study population includes a racially and ethnically diverse group of 3,939 men and women aged 21–74 years with mild to moderate renal disease with an estimated glomerular filtration rate (eGFR) of 20–70 ml/min/1.73 m^2^ and about half of whom have diabetes. Phase-1 CRIC participants were recruited between May 2003 and August 2008 from seven centers in the United States. Patients were identified through laboratory database searches of recently measured serum creatinine values, referrals from physicians’ offices, and self-referral. Patients with cirrhosis, HIV infection, polycystic kidney disease, renal cell carcinoma, a kidney transplant or on dialysis, or taking immunosuppressant drugs were excluded. Cardiovascular events were adjudicated using a standard Medical Event Questionnaire during all follow-up interviews. To align with the CPROBE outcome, CVD in CRIC was defined as incident cases of CHF, acute MI, stroke, and peripheral arterial disease (CVDCOMP4). For participants who were hospitalized owing to a CVD event, their medical records were requested for verification. Two physicians adjudicated each cardiovascular event for concordant ascertainments. Upon discordance between two adjudicators, and after adequate discussion, a third independent adjudicator assessed the outcome until concordance achieved. MI was based on symptoms of angina, cardiac biomarkers, and electrographic data. CHF was based on hospital admission for new or worsening heart failure signs and symptoms and diminished cardiac output. Ischemic stroke was defined as a physician-ascertained, patient-reported prior episodes of central nervous system infarction diagnosed based on clinical evidence, imaging, or other objective evidence (26). The CRIC samples in the current study is a case-control observation nested in the phase-1 parent CRIC. As of October 2019, out of 3,939 participants from phase-1 of the parent CRIC, 1,255 (31.9%) developed CV outcomes, a rate that is comparable with parent CPROBE cohort. For this sub-study, we analyzed a total of 91 baseline samples from participants without clinically apparent CVD: 34 who had an incident CVD event during follow-up and 57 who did not. In contrast to the CPROBE samples, the participants in CRIC were not matched by any parameters. The goal of analyzing the CRIC samples was to validate the findings in CPROBE cohort. The analysis of the samples was approved by the CRIC Consortium at the University of Michigan. All samples were deidentified and analyzed in a blinded manner for cholesterol efflux and HDL particle measurements.

The human studies reported in this manuscript abide by the Declaration of Helsinki principles.

### Sample storage

The plasma samples from each CKD cohort were stored frozen at −80°C without cryoprotectants for extended and various lengths of time, up to 12 years in the CPROBE cohort and up to 15 years for the CRIC cohort. It has been shown that HDL structure and function is profoundly affected when isolated HDL is stored frozen in the absence of cryoprotectants (27). To address whether storage alters HDL-P and/or CEC, we performed analyses of plasma samples from 179 subjects, a subset of subjects randomly selected from participants with or without severe carotid cerebrovascular disease and with or without diabetes enrolled in the CLEAR study (28) for sample stability analysis upon extended storage time. These plasma samples were collected over more than 15 years without cryoprotectants for sample storage. Serum HDL was freshly isolated from the stored samples for measurement of CEC and concentrations of different-sized HDL particles, like in the CRIC and CPROBE studies. Unlike storage of isolated HDL (27), there was no significant impact of sample storage time on serum HDL-CEC or the concentration of medium-sized HDL-P (supplemental Fig. S1). Therefore, the different lengths of plasma storage time are unlikely to affect the HDL measurements. These observations also suggest that HDL is much more stable when stored in plasma samples than as isolated HDL.

### Laboratory measurements

Hypertension was defined as systolic blood pressure >140 mm Hg, diastolic blood pressure >90 mm Hg, or the use of antihypertensive drugs. Standard enzymatic methods were used to measure lipid profiles. eGFR was calculated using the CKDEPI equation for adults from serum creatinine measured on an ADVIA 2400 analyzer using the Jaffe reaction. Glomerular disease included diabetic kidney disease, focal segmental glomerulosclerosis, membranous glomerulopathy (membranous nephropathy), IgA nephropathy, vasculitis, immune-complex glomerulonephritis, and lupus nephritis.

### Assessing HDL’s CEC

The ability of serum HDL to promote cholesterol excretion was assessed using cAMP-stimulated J774 macrophages as previously described (29, 30, 31). The goal of these measurements was to determine whether the CEC of HDL isolated from CKD patients was associated with incident CVD events.

### HDL-P and size measurement

**Fig. 1:**
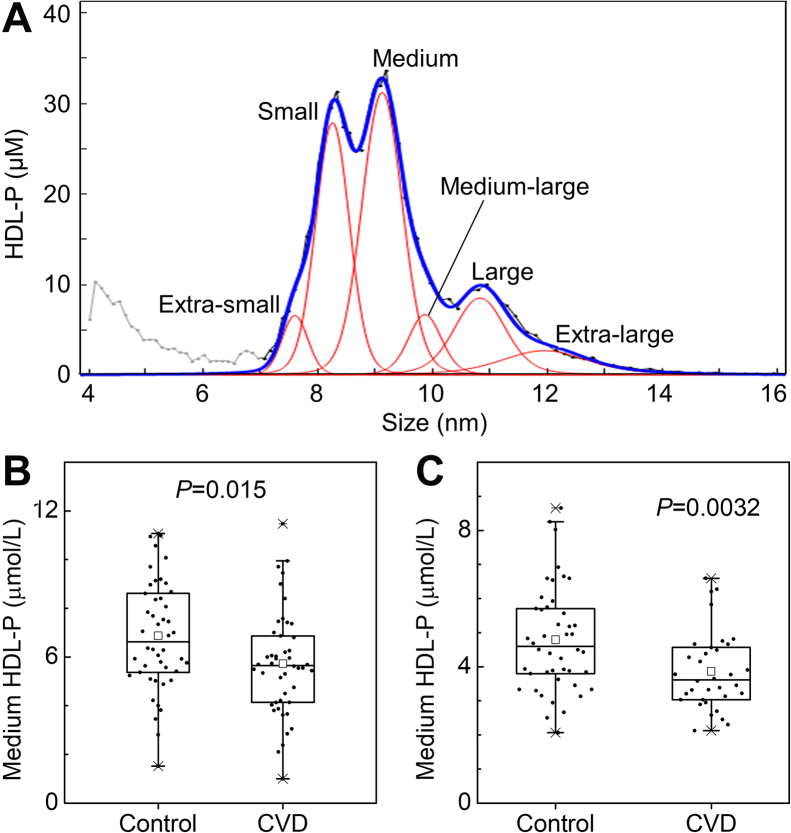
Calibrated ion mobility analysis of six HDL particle (HDL-P) populations and quantification of medium-sized HDL-P in CKD patients with or without incident CVD. A: HDL-P profile measured by calibrated IMA on a differential mobility analyzer (DMA). Six HDL subspecies (extra-small, ∼7.6 nm; small, ∼8.3 nm; medium, ∼9.1 nm; medium-large, ∼9.7 nm; large, ∼10.8 nm; and extra-large, ∼12.3 nm) were fitted to the DMA profiles by unsupervised, iterative curve fitting. B: Quantification of medium HDL-P in CPROBE cohort. C: Quantification of medium HDL-P in CRIC cohort. The box plots show the distribution of the data of medium HDL-P (median, interquartile ranges), while the dots represent individual data points. Crosses (x) represent 99% and 1% levels. The small squares within the boxes represent mean levels. *P*-values are from a Mann-Whitney U test. CKD, chronic kidney disease; CPROBE, Clinical Phenotyping and Resource Biobank Core; CRIC, Chronic Renal Insufficiency Cohort; IMA, ion mobility analysis.

HDL-P was quantified by cIMA on a differential mobility analyzer (DMA) (TSI Inc., Shoreview, MN), as previously reported (21). In brief, total lipoproteins were isolated from plasma by a single ultracentrifugation (density 1.21 g/ml) and then dialyzed to remove salts (which interfere with IMA). After the samples were diluted, HDLs were separated according to size by DMA and detected by a laser light-scattering particle counter. Six HDL subspecies (extra-small, ∼7.6 nm; small, ∼8.3 nm; medium, ∼9.1 nm; medium-large, ∼9.7 nm; large, ∼10.8 nm; and extra-large, ∼12.3 nm, Fig. 1A) were fitted to the DMA profiles by unsupervised, iterative curve fitting. Areas under the curve fitted for each subspecies were directly converted to HDL-P using a glucose oxidase calibration curve. The % CVs were <15% for HDL subpopulations, with the exception of small HDL (20.4%).

### Statistical analysis

Baseline demographic and clinical characteristics were compared using a two-tailed unpaired Student’s *t* test for normally distributed data or the Mann-Whitney nonparametric U test for abnormally distributed data. We used the Shapiro-Wilk tests to assess the normality of distribution.

Categorical variables were compared using the chi-square test to compare frequencies between groups. Univariate and multivariate models were built for the HDL metrics using logistic regression analysis with incident CVD as a dependent variable, and the results were expressed in odds ratios (ORs) with a 95% confidence interval (CI). The logistic regression models were adjusted for potential clinical confounders, including age, gender, diabetes status, hypertension, present smoker, body mass index (BMI), and eGFR (gender and diabetes status were matched for the CPROBE cohort). The models were further adjusted for levels of lipids (including LDL-C, triglycerides, and HDL-C). Finally, the models were further adjusted for C-reactive protein (CRP) and urinary protein to creatinine (UPC) ratio for CPROBE cohort and UPC ratio for CRIC cohort. ORs were reported per 1-SD increment of levels of HDL metrics. All statistical analyses were performed using SPSS (Windows version 19, Chicago, IL).

## Results

The clinical characteristics of the 92 CKD subjects (46 controls without CVD outcomes and 46 with incident CVD) in CPROBE are listed in Table 1. We matched the two groups (control and incident CVD) by gender and diabetes status. We also roughly matched age (within 10 years for matched pairs), percentages of individuals identifying as whites, and CKD stages (or levels of eGFR) for the matched pairs. The two groups had similar levels of HDL-C, LDL-C, total cholesterol, triglycerides, CRP, serum creatinine, systolic blood pressure, and diastolic blood pressure. The incident CVD group had significantly higher UPC ratios.

**Table 1:** Clinical characteristics of CPROBE subjects

Covariate	Control	Incident CVD	*P*-value
Number of subjects	46	46	
Age (years)	55.3 (31–79)	54.2 (31–81)	0.68^a^
Gender (female)	31 (67.4)	31 (67.4)	1.00
BMI (kg/m^2^)	31.9 (27.2–37.1)	33.3 (27.5–39.2)	0.44^b^
White	31 (67.4)	33 (71.7)	0.64
SBP (mmHg)	133.0 (121–140)	135.5 (118–147.8)	0.55^b^
DBP (mmHg)	71.0 (65–77)	73.0 (65.5–84)	0.22^b^
Hypertension	33 (71.7)	35 (76.1)	0.64
Ever a smoker	18 (39.1)	16 (34.8)	0.67
Present Smoker	5 (10.9)	7 (15.2)	0.54
Diabetes	16 (34.8)	16 (34.8)	1.00
Proteinuria	4 (8.7)	9 (19.6)	0.14
Glomerular disease	16 (34.8)	15 (32.6)	1.00
Serum creatinine (mg/dl)	1.4 (1.1–1.7)	1.6 (1.0–2.3)	0.45^b^
eGFR (mL/min/1.73 m^2^)	44.3 (33.6–70.2)	43.9 (26.9–79.0)	0.53^b^
CKD stage			
1 & 2 (eGFR ≥ 60)	15 (32.6)	16 (34.8)	0.83
3 (30 ≤ eGFR < 60)	21 (45.7)	12 (26.1)	0.050
4 & 5 (eGFR < 30)	10 (21.7)	18 (39.1)	0.070
UPC ratio	0.91 (0.19–3.49)	2.86 (0.54–5.65)	<0.0001^b^
CRP (mg/dl)	0.315 (0.080–0.623)	0.450 (0.146–1.300)	0.058^b^
Statin use	26 (56.5)	18 (39.1)	0.095
HDL-C (mg/dl)	46 (39–56.8)	45.5 (35.5–51.5)	0.24^a^
LDL-C (mg/dl)	98.9 (75.5–113.4)	108.3 (86.6–123.3)	0.14^b^
Total cholesterol (mg/dl)	181 (163.5–201.8)	196 (168.3–219.3)	0.14^b^
Triglycerides (mg/dl)	146.5 (120.5–212)	199 (123–238)	0.19^b^
Serum HDL CEC (%)	10.29 (8.50–12.53)	10.08 (8.54–12.26)	0.96^b^

Entries are median (IQR) for continuous covariates and N (%) for categorical covariates. We matched cases to controls for gender and diabetic status in a one-to-one ratio. *P*-values are from a Student’s *t* test for normally distributed variables (a), a Mann-Whitney U test for abnormally distributed variables (b), or a chi-square test for categorical variables. Shapiro-Wilk test was used for the normality test.

BMI, body mass index; CEC, cholesterol efflux capacity (J774+cAMP); CKD, chronic kidney disease; CRP, C-reactive protein; DBP, diastolic blood pressure; eGFR, estimated glomerular filtration rate; SBP, systolic blood pressure; UPC ratio, Urinary protein to creatinine ratio.

Using the same samples from the CPROBE cohort, we have reported that levels of four proteins in HDL—APOA4, LCAT, PON1, and PON3—were negatively associated with incident CVD events in patients with CKD (23). In the current study, we assessed whether other HDL metrics, including HDL-C, serum HDL-CEC, and various sized HDL-P also associate with incident CVD in CKD patients. We did not match the levels of HDL-C in the CPROBE samples, but the levels of HDL-C were similar in both groups (Table 1). To determine whether HDL’s CEC associates with future CVD events in subjects with CKD, we compared the CEC of serum HDL isolated from the control and CVD groups. The two groups did not differ significantly (10.29% vs. 10.08%; control vs. CVD, *P* = 0.96) (Table 1). To determine whether HDL-P associate with future CVD events, we measured the concentrations of different-sized HDL particles, including extra-small, small, medium, medium-large, large, and extra-large by cIMA. Representative profiles of different-sized HDL-P are shown in Fig. 1A. Levels of medium HDL particles were significantly lower in the CVD group than in the control group in the CPROBE cohort (5.65 μM vs. 6.62 μM, *P* = 0.015) (Fig. 1B and supplemental Table S1). No significant differences in concentration were found for other-sized HDL particles or in the concentration of total HDL-P or in size distribution within each HDL subpopulation (supplemental Table S1).

**Fig. 2:**
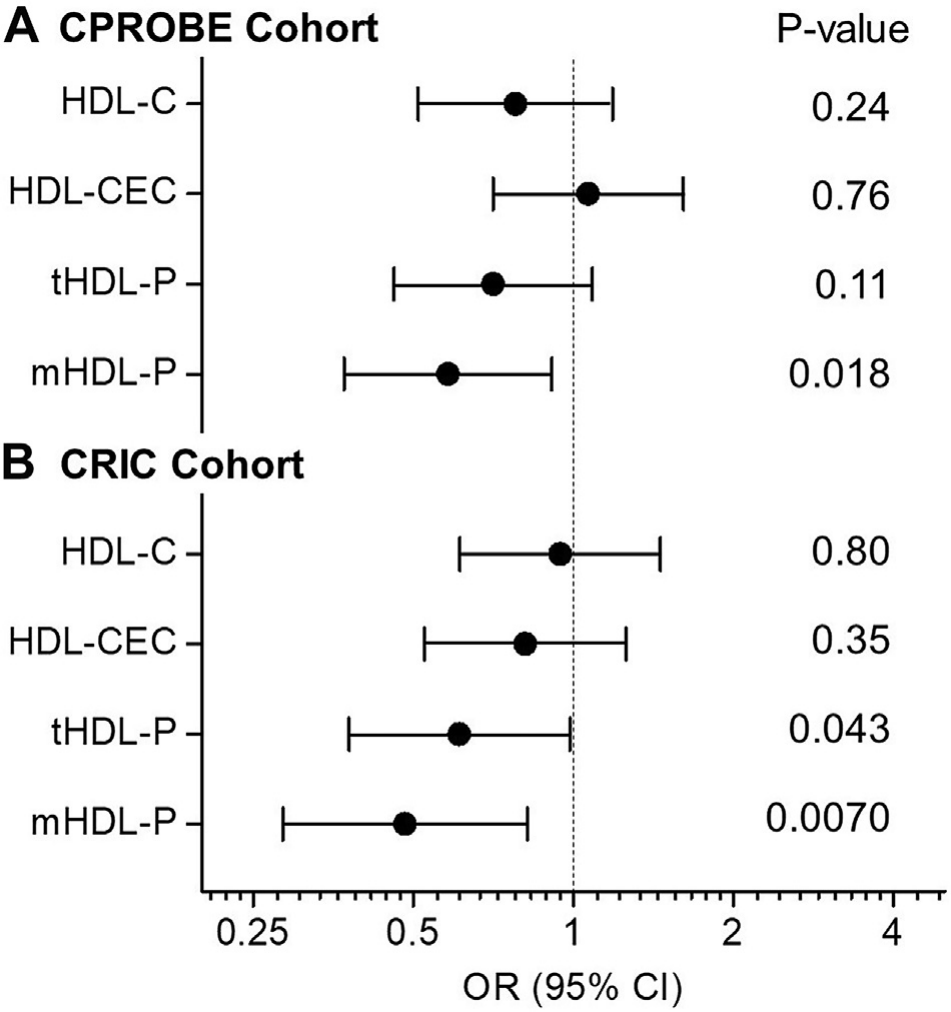
Odds Ratio of HDL metrics for incident CVD in CKD. Unadjusted odds ratios and *P*-values were obtained from univariate logistic regression analysis. The levels of HDL cholesterol (HDL-C), HDL’s cholesterol efflux capacity (CEC), or HDL particle concentration (HDL-P) were used as an independent variable in the logistic regression analysis, and incident CVD was the outcome. Odds ratios are per SD increase of HDL metrics. A: CPROBE cohort (N = 46 for control group and N = 46 for incident CVD group). B: CRIC cohort (N = 57 for control group and N = 34 for incident CVD group). CKD, chronic kidney disease; CPROBE, Clinical Phenotyping and Resource Biobank Core; CRIC, Chronic Renal Insufficiency Cohort; mHDL-P, medium HDL-P; tHDL-P, total HDL-P.

**Fig. 3:**
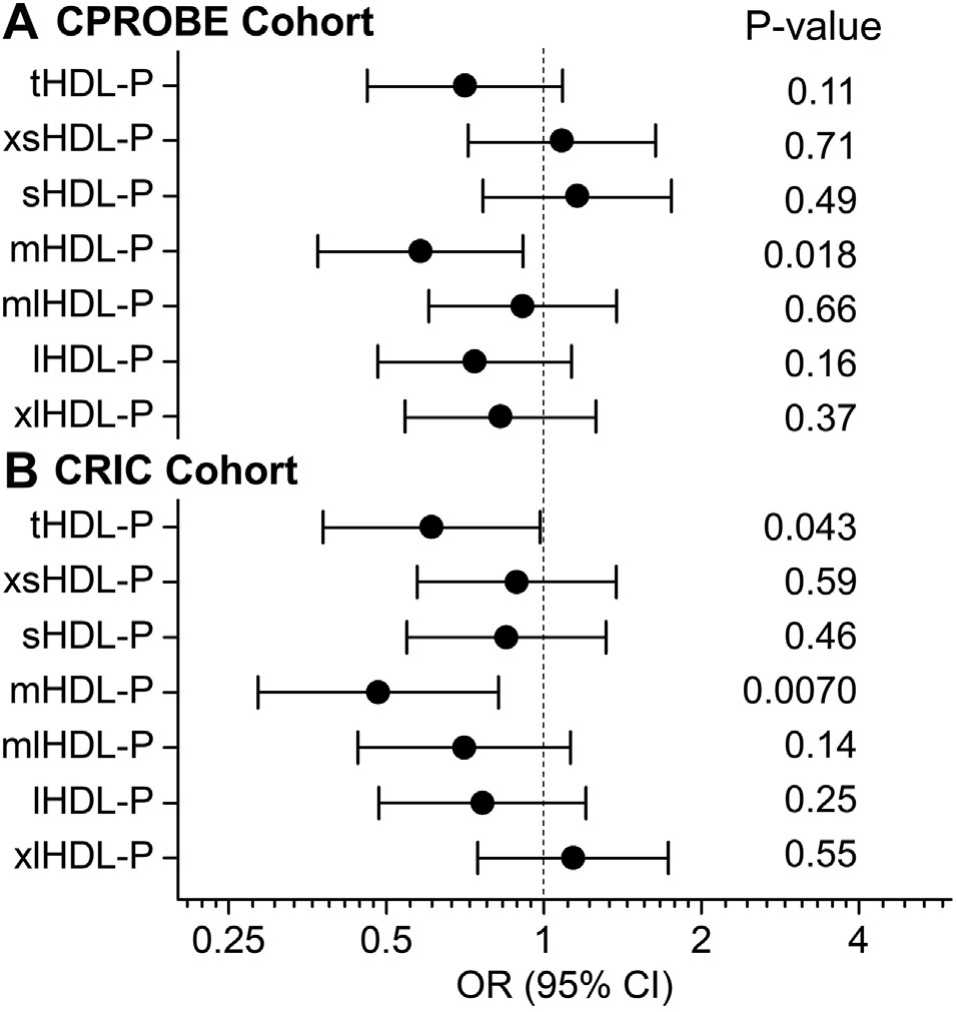
Odds Ratio of total HDL-P and different-sized HDL-P for incident CVD in CKD. Unadjusted odds ratios and *P*-values of total HDL-P and different-sized HDL-P were obtained from univariate logistic regression analysis. The levels of different HDL-P were used as an independent variable in the logistic regression analysis, and incident CVD was the outcome. Odds ratios are per SD increase of HDL-P. A: CPROBE Cohort (N = 46 for control group and N = 46 for incident CVD group). B: CRIC cohort (N = 57 for control group and N = 34 for incident CVD group). CKD, chronic kidney disease; CPROBE, Clinical Phenotyping and Resource Biobank Core; CRIC, Chronic Renal Insufficiency Cohort; HDL-P, HDL-Particle concentration; lHDL-P, large HDL-P; mHDL-P, medium HDL-P; mlHDL-P, medium-large HDL-P; sHDL-P, small HDL-P; tHDL-P, total HDL-P; xlHDL-P, extra-large HDL-P; xsHDL-P, extra-small HDL-P.

**Fig. 4:**
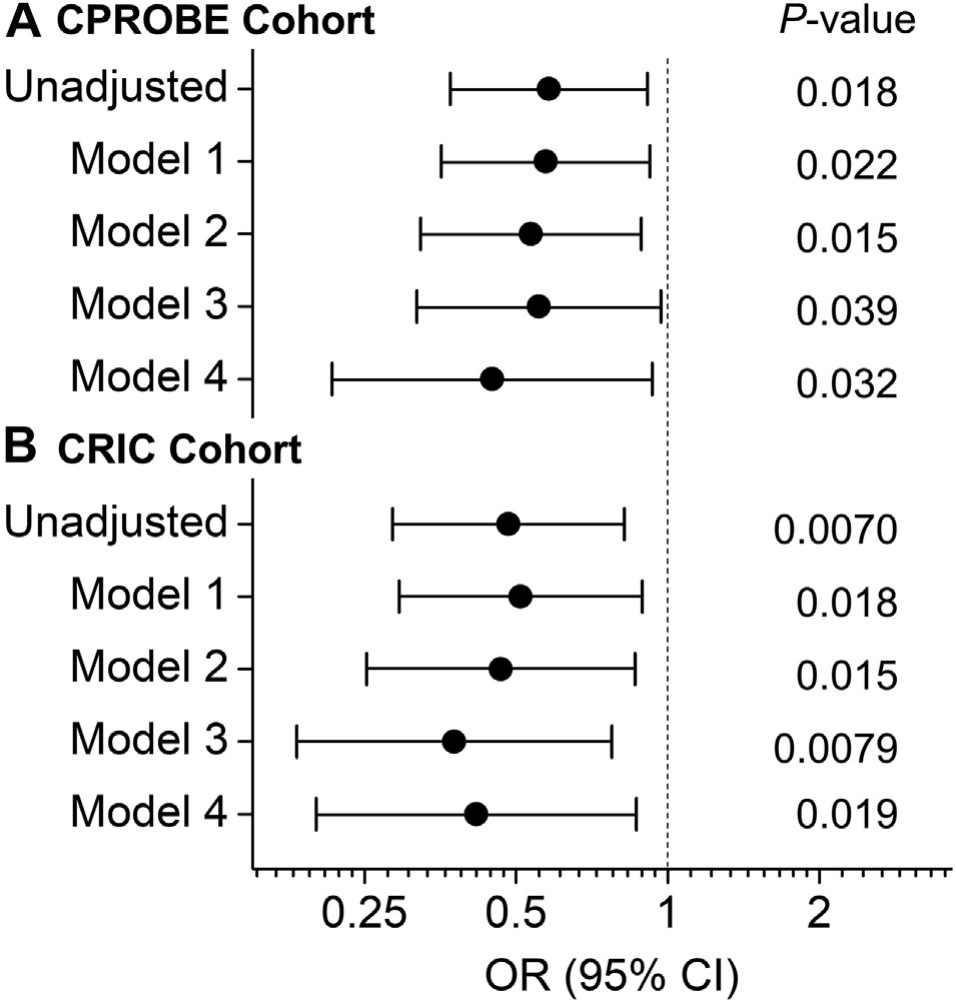
Odds Ratio of medium HDL-P for incident CVD in CKD. Unadjusted and adjusted odds ratios (ORs, the solid circle dot), 95% confidence interval (CI), and *P*-values were obtained from univariate and multivariate logistic regression analysis. The levels of medium HDL-P (mHDL-P) were used as an independent variable in the logistic regression analysis, and incident CVD was the outcome. Odds ratios are per SD increase of levels of mHDL-P. A: CPROBE Cohort (N = 46 for control group and N = 46 for incident CVD group). Model 1 is a model adjusted for age and hypertension. Model 2 is model 1 adjusted for additional potential clinical confounders including present smoker, BMI, and eGFR. Model 3 is model 2 further adjusted for lipid risk factors including LDL-C, log-transformed TGs, and HDL-C. Model 4 is model 3 further adjusted for C-reactive protein (CRP) and urinary protein to creatinine ratio (UPR ratio). B: CRIC cohort (N = 57 for control group and N = 34 for incident CVD group). Model 1 is a model adjusted for age, hypertension, sex, and diabetes. Model 2 is model 1 adjusted for additional potential clinical confounders including present smoker, BMI, and eGFR. Model 3 is model 2 further adjusted for lipid risk factors including LDL-C, TGs, and HDL-C. Model 4 is model 3 further adjusted for urinary protein to creatinine ratio (UPR ratio). BMI, body mass index; CKD, chronic kidney disease; CPROBE, Clinical Phenotyping and Resource Biobank Core; CRIC, Chronic Renal Insufficiency Cohort; eGFR, estimated glomerular filtration rate; HDL-P, HDL-Particle concentration; TG, triglyceride.

Next, we used logistic regression analysis to assess the association of HDL metrics with incident CVD. First, we obtained unadjusted ORs for HDL-C, HDL-CEC, total HDL-P, and different-sized HDL-P in predicting incident CVD. As shown in Fig. 2A, Fig. 3A, only the levels of medium HDL-P were associated significantly and negatively with incident CVD. In contrast, the levels of HDL-C, HDL-CEC, total HDL-P, or other-sized HDL-P did not associate with incident CVD. Next, we adjusted the model of medium HDL-P for age and hypertension. The diabetic status and sex were not included in the models because the subjects were matched one-to-one by diabetic status and sex. The differences in the concentrations of medium HDL-P remained significant and negative association with incident CVD after adjustment for age and hypertension (Fig. 4A model 1). Then we further adjusted model 1 for additional clinical confounders, including present smoker, BMI, and eGFR. The analysis demonstrated that the association between the levels of medium HDL-P and incident CVD was still significant (Fig. 4A model 2). When we further added lipid risk factors, including LDL-C, log-transformed triglycerides, and HDL-C into model 2, with an OR of 0.55 (95% CI 0.32–0.97), the association between medium HDL-P and incident CVD remained significant (*P* = 0.039) (Fig. 4A model 3). Finally, when we further adjusted for CRP and UPC ratio, the association between medium HDL-P and incident CD was still significant (*P* = 0.032), and the OR was decreased to 0.45 (95% CI 0.22–0.93). It is important to note that, in the multivariate analyses, except for UPC in the final model, no other adjusted confounders were associated with incident CVD (supplemental Table S2).

In order to verify our observations on the negative association of medium HDL-P with CVD in the CPROBE subjects, we analyzed another set samples from the CRIC cohort. The clinical characteristics of the 91 CKD subjects (57 controls without CVD outcomes and 34 with incident CVD) are listed in Table 2. The two groups had similar levels of HDL-C, LDL-C, total cholesterol, triglycerides, serum creatinine, and systolic blood pressure. The incident CVD group had significantly higher UPC ratio. The CVD group in CRIC samples had slightly but significantly higher diastolic blood pressure and significantly higher number of patients with end-stage renal disease (22 in the CVD group vs. 11 in the control group).

**Table 2:** Clinical characteristics of CRIC subjects

Covariate	Control	Incident CVD	*P*-value
Number of subjects	57	34	
Age (years)	60 (53–64.5)	58.5 (46.8–65.3)	0.16^a^
Gender (female)	28 (49.1)	12 (35.3)	0.20
BMI (kg/m^2^)	30.9 (27.2–36.1)	32.3 (27.6–36.6)	0.96^b^
White	28 (49.1)	17 (50.0)	0.94
SBP (mmHg)	124.0 (114.3–139.3)	129.0 (113.2–145.7)	0.18^a^
DBP (mmHg)	71.3 (60.0–79.3)	73.3 (66.0–82.7)	0.039^a^
Hypertension	49 (86.0)	32 (94.1)	0.23
Ever a smoker	35 (61.4)	15 (44.1)	0.11
Present Smoker	9 (15.8)	5 (14.7)	0.89
Diabetes	34 (59.6)	21 (61.8)	0.84
eGFR (mL/min/1.73 m^2^)	43.1 (35.5–55.2)	43.3 (34.8–50.7)	0.71^b^
Serum creatinine (mg/dl)	1.70 (1.40–1.90)	1.75 (1.50–2.13)	0.23^a^
Serum albumin (g/dl)	4.1 (3.8–4.4)	4.0 (3.6–4.3)	0.052^a^
UPC ratio	0.13 (0.052–0.45)	0.65 (0.12–1.84)	0.0013^b^
Statin use	30 (52.6)	17 (50.0)	0.74
HDL-C (mg/dl)	46.5 (36.8–56.5)	47.0 (35.5–52.8)	0.53^b^
LDL-C (mg/dl)	96.5 (76.8–123.5)	101.5 (80.8–122.5)	0.88^b^
Total cholesterol (mg/dl)	177 (151.8–201.5)	178 (166–218)	0.31^b^
Triglycerides (mg/dl)	122.5 (82.3–170)	146 (98.8–194)	0.19^b^
Serum HDL CEC (%)	10.25 (8.83–11.76)	9.49 (7.58–11.80)	0.36^a^

Entries are median (IQR) for continuous covariates and N (%) for categorical covariates. *P*-values are from a Student’s *t* test for normally distributed variables (a), a Mann-Whitney U test for abnormally distributed variables (b), or a Pearson chi-square test for categorical variables. Shapiro-Wilk test was used for the normality test.

BMI, body mass index; CEC, cholesterol efflux capacity (J774+cAMP); DBP, diastolic blood pressure; eGFR, estimated glomerular filtration rate; SBP, systolic blood pressure; UPC ratio, urinary protein to creatinine ratio.

The levels of HDL-C were similar in both groups in the CRIC cohort (Table 2). We compared the CEC of serum HDL isolated from the control and CVD groups for the CRIC samples. The two groups did not differ significantly (10.25% vs. 9.49%; control vs. CVD, *P* = 0.36) (Table 2). We measured the concentrations of total HDL-P and different-sized HDL-P. Levels of total HDL-P and medium HDL-P were significantly lower in the CVD group than in the control group (12.27 μM vs. 9.60 μM, *P* = 0.0073 for total HDL-P and 4.51 μM vs. 3.62 μM, *P* = 0.0032 for medium HDL-P) (supplemental Table S3 and Fig. 1C). No significant differences in concentration were found for other-sized HDL particles or sizes of HDL particles within the different HDL subpopulations.

We then obtained unadjusted ORs using logistic regression analysis for HDL-C, HDL-CEC, total HDL-P, and size distribution of the different HDL subpopulations in predicting incident CVD in CRIC. As shown in Figs. 2B, 3B, the lower concentrations of total HDL-P and medium HDL-P were associated significantly with incident CVD (OR = 0.61, *P* = 0.043 and OR = 0.48, *P* = 0.0070 for total and medium HDL-P, respectively). In contrast, the levels of HDL-C, HDL-CEC, or other-sized HDL-P did not associate with incident CVD. Next, we adjusted the model of medium HDL-P for age, hypertension, sex, and diabetes. The decreased concentrations of medium HDL-P remained significant and negative association with incident CVD (Fig. 4B model 1). Then we further adjusted model 1 for additional clinical confounders, including present smoker, BMI, and eGFR. The analysis demonstrated that the association between the levels of medium HDL-P and incident CVD was still significant (Fig. 4B model 2). When we further added lipid risk factors, including LDL-C, log-transformed triglycerides, and HDL-C into model 2, with an OR of 0.38 (95% CI 0.18–0.77), the association between medium HDL-P and incident CVD was still significant (*P* = 0.0079) (Fig. 4B model 3). Finally, when we further adjusted the models for UPC ratio, the association between medium HDL-P and incident CVD remained significant (*P* = 0.019) with an OR of 0.42 (95% CI 0.20–0.87) (Fig. 4B model 4). In contrast to the CPROBE cohort, in model 3 of multivariate analyses, log-transformed triglyceride and HDL-C were significantly associated with incident CVD; while in the complete model of multivariate analyses (model 4), no adjusted confounders (including UPC ratio) were significantly associated with incident CVD (supplemental Table S4). It is noteworthy that HDL-C was positively associated with incident CVD in the final model (model 4) when UPC ratio was included in the model in the CRIC cohort.

## Discussion

Using cIMA, we measured the concentrations of six different-sized HDL subpopulations and the size distribution within each HDL subpopulation. Our observations demonstrate that lower concentrations of medium HDL-P, but not concentrations of other-sized HDL particle subpopulations, are associated with incident CVD in two independent clinical cohorts (CPROBE and CRIC) after adjusting for traditional clinical confounders and lipid risk factors. Although lower concentrations of total HDL-P were also associated with incident CVD in the univariate analysis and in two of the three adjusted models in CRIC participants, total HDL-P did not predict incident CVD in the CPROBE cohort. In either cohort, there was no association of HDL particle size distribution within the different HDL subpopulations or of HDL-C with incident CVD. We also measured serum HDL-CEC as the ability of APOB-depleted serum to elicit cholesterol efflux from cAMP-stimulated J774 macrophages. Our observations revealed that serum HDL-CEC was not associated with incident CVD in either cohort. Taken together, among the HDL metrics we assessed in the current study, our observations indicate that only lower levels of medium-sized HDL-P—but not concentrations of other-sized HDL particles, total HDL-P, HDL-C, or HDL-CEC—associate with incident CVD in two separate cohorts of patients with CKD independent of the traditional cardiovascular risk factors.

The failure of several clinical trials elevating HDL-C to reduce cardiovascular risk have cast doubt on whether high levels of HDL-C are cardioprotective (6). However, clinical and epidemiological studies show robust negative association of HDL-C levels with CVD risk in the general population (3). Indeed, a meta-analysis of 68 long-term prospective studies including >300,000 participants free of CVD at baseline and a follow-up time of 2.79 million person-years revealed that each 1-SD increase (i.e., 15 mg/dl) in HDL-C was associated with a 29% risk reduction after adjusting for nonlipid risk factors and a 22% risk reduction after further adjusting for non-HDL cholesterol and log-transformed triglycerides (32). Importantly, low HDL-C associates with poor kidney function and progression of renal disease (22). It is unclear, however, whether low HDL-C contributes to increased CVD risk in patients with CKD. Interestingly, a 3-year study of 33,109 CKD patients on hemodialysis revealed a U-shaped association between HDL-C levels and the risk of total and CVD mortality (33). Thus, HDL-C concentrations up to 50 mg/dl were associated with better overall survival in hemodialysis patients, while HDL-C at 60 mg/dl and above was associated with increased all-cause and CVD mortality. In the current study, HDL-C levels did not associate with incident CVD either in the CPROBE cohort or in the CRIC cohort.

Recent findings suggest that functional properties of HDL, especially the CEC of serum HDL, might better reflect its cardioprotective properties. In the general population with normal kidney function and among individuals at elevated cardiovascular risk, the CEC of serum HDL (HDL-CEC) associates negatively with incident CVD after adjustment for HDL-C levels and other clinical confounders (10, 11). It is important to note that HDL isolated from CKD and dialysis subjects has impaired CEC compared with HDL from control subjects (34, 35). These observations suggest that impaired renal function could promote CVD by diminishing HDL’s CEC. However, in the CARE FOR HOMe study of patients with CKD stages 2–4, HDL-CEC was not significantly different between patients with and without prevalent CVD at baseline, and it did not predict CVD events that occurred during the prospective 4.6 years of observation period (36). In another study, the German Diabetes Dialysis Study (the 4D-study) of diabetic subjects on hemodialysis, HDL-CEC did not predict cardiovascular risk over a median follow-up of 4.1 years (37). In agreement with the above two studies, in the current study, we did not find a significant association between HDL-CEC and incident CVD events in the two cohorts of CKD patients. Moreover, one large study of a cohort free of known CVD at baseline revealed that higher CEC may actually predict an increased CVD risk in CKD patients, while CEC was negatively associated with incident CVD in subjects without CKD (38). Our observations together with previous studies indicate that unlike in the general population, impaired CEC is not a prognostic cardiovascular risk marker in patients with CKD.

HDL-P represent another HDL metrics that could serve as a prognostic cardiovascular risk marker. Multiple large-scale human studies revealed that diminished HDL-P can be superior to impaired HDL-CEC or reduced HDL-C levels for predicting future CVD events. In the JUPITER Trial (Justification for the Use of Statins in Prevention: An Intervention Trial Evaluating Rosuvastatin) (13, 39), HDL-CEC was negatively associated with incident CVD in individuals on potent statin therapy but not at baseline. However, for both baseline and on-statin treatment, total HDL-P was the strongest of four HDL-related biomarkers (HDL-P, HDL-CEC, HDL-C, and APOA1) as a negative predictor of incident CVD events and biomarker of residual risk. In Multi-Ethnic Study of Atherosclerosis, elevated total HDL-P but not HDL-C was associated with reduced risk of incident CVD events and decreased carotid intima-media thickness after adjusting for each other and LDL particle number for subjects without coronary heart disease at baseline or lipid altering therapy (40). In agreement, European Prospective Investigation into Cancer and Nutrition-Norfolk, a study on apparently healthy subjects with 6 years of follow-up, showed that a high total HDL-P predicted reduced risk of coronary heart disease following multiple adjustments (16).

Moreover, recent studies have revealed that HDL subpopulations show different degrees of association with CVD risk. Among different techniques used to separate and measure HDL subpopulations (14), NMR is the major technique used in most studies on HDL-P. HDL particles of different size may vary in their composition and functions (41). There is currently no consensus on which subpopulation of HDL is most beneficial, but medium HDL-P has been frequently suggested to be protective. For example, in the Heart Protection Study, medium HDL-P was the strongest predictor of reduced coronary events in statin treated subjects, compared with large and small HDL-P (42). Likewise, in the Multiple Risk Factor Intervention Trial, medium-sized HDL-P predicted reduced risk of coronary heart disease death after adjustment for traditional risk factors. In contrast, large- or small-sized HDL-P or total HDL-P, HDL-P size, or HDL-C was not related to risk of coronary heart disease death in that study (43).

All the above-mentioned studies investigating the relationship between HDL-P and incident CVD risk were centered on subjects with normal kidney function. In one study in which a subset of participants had baseline CKD, the authors demonstrated that total HDL-P was negatively associated with incident CVD independent of CKD status (38). That study provided no information on different HDL subpopulations. In the current study, using cIMA, we measured concentrations of six different HDL subpopulations (extra-small, small, medium, medium-large, large, and extra-large HDL-P) in two prospective cohorts of CKD patients. Our results demonstrated that, in both the CPROBE and CRIC cohorts, medium HDL-P—but not other-sized HDL-P or total HDL-P—significantly and negatively associated with incident CVD in subjects with CKD in all models adjusted for clinical confounders and lipid risk factors, including HDL-C.

One strength of our study is that we analyzed samples in CPROBE and CRIC, two independent and prospective cohorts of CKD patients. We demonstrated that only medium-sized HDL-P predicts incident CVD in both cohorts. Another strength of our study is that the sizes and the concentrations of different HDL particles were measured by cIMA, a well validated method that can directly and accurately measure and quantify different-sized HDL particles (21). A limitation of our study is the relatively small sample size of both cohorts. It will be essential to confirm and extend our findings with larger cohorts of CKD subjects with longer follow-up. Another limitation of our approach is that association does not prove causality. Future studies will need to investigate the molecular mechanisms whereby medium-sized but not other-sized HDL-P protects against atherosclerosis in CKD and if elevating its levels will reverse CKD-associated atherosclerosis in model systems.

It is important to point out that, although cIMA is more accurate than NMR in measuring concentrations of HDL subpopulations, IMA is currently not ready to be established in clinical laboratories because it requires a single ultracentrifugation and a dialysis step before analysis, which is laborious (see Materials and Methods section for detail). HDL particle sizes and concentrations measured by IMA agree poorly with the measurements by NMR (14), which is widely used in clinical studies to quantify HDL particle number because of its ability to quantify a variety of lipoproteins including their subclasses rapidly and cost-effectively (44). Because the same samples used in the current study were not analyzed by NMR, we cannot compare HDL measurements using the two methods. Nevertheless, absolute accuracy aside, relative comparisons between samples using the same methodology (either by IMA or NMR) can be clinically useful.

It is also important to note that medium-sized HDLs are a highly heterogeneous mixture, consisting of several particle species whose properties, such as proteome, lipidome, and functions, may vary substantially. Moreover, they may vary to a marked degree in their structural and functional features between individuals exhibiting different disorders and even between individuals with the same disorder. Therefore, in order to investigate the cardioprotective mechanism of different-sized HDL species, further isolation and characterization of individual HDL particles will be needed (45, 46, 47).

What could be the mechanism of medium-sized HDL’s protective effects on CVD, if there is indeed a causal association? Our study suggests that the cardioprotective function of medium-sized HDL is unlikely to be explained by improved CEC. Consistent with the present study, it has been shown that elevated concentrations of medium-sized HDL particles are associated with protection from the vascular complications of type 1 diabetes, while the ABCA1-specific CEC of serum HDL and the CEC by J774 macrophages were not different between subjects with and without vascular complications (48). Previous studies also demonstrated that small HDL is more efficient than larger HDL subpopulations in promoting cholesterol efflux from cells (46, 49). It is possible that while small-sized HDL is better at cholesterol efflux from macrophages, larger HDLs may be better at protecting the endothelium (50). Another explanation is perhaps the different composition of HDL subpopulations. For example, we have recently shown that HDL-associated PON1, an enzyme with athero-protective properties (51, 52), is positively correlated with medium-sized HDL-P and protection from vascular complications in subjects with type 1 diabetes (48). Importantly, we demonstrated that HDL-associated PON1 is negatively associated with incident CVD events in CPROBE (23). Whether PON1 or other proteins carried by medium-sized HDL mediate its atheroprotection needs further study.

In conclusion, we demonstrate that concentrations of medium-sized HDL-P—but not other-sized HDL-P or total HDL-P, the sizes within each HDL subpopulation, HDL-C, or HDL-CEC—were negatively associated with incident CVD events in both CPROBE and CRIC, two prospective cohorts of patients with CKD. Importantly, medium HDL-P was significantly associated with CVD protection in all three adjusted models, indicating that the associations were independent of clinical characteristics and lipid risk factors, including HDL-C. Considering that HDL-P is superior to HDL-CEC and HDL-C as a prognostic cardiovascular risk marker, medium-sized HDL-P may represent a therapeutic target for CVD in CKD patients.

## Data availability

The data supporting this study are available in the article or available from the corresponding author upon reasonable request.

## Supplemental data

This article contains supplemental data (28).

## Conflict of interest

The authors declare that they have no conflicts of interest with the contents of this article.
