# Supplementary material for: Low concentrations of medium-sized HDL particles predict incident CVD in chronic kidney disease patients
Source: J Lipid Res. 2023 Apr 24;64(6):100381. doi: 10.1016/j.jlr.2023.100381 (PMC10323925; doi:10.1016/j.jlr.2023.100381)
Supplement: Supplemental Figure S1 and Tables 1–4 [file mmc1.pdf]

Supplemental Figure 1.

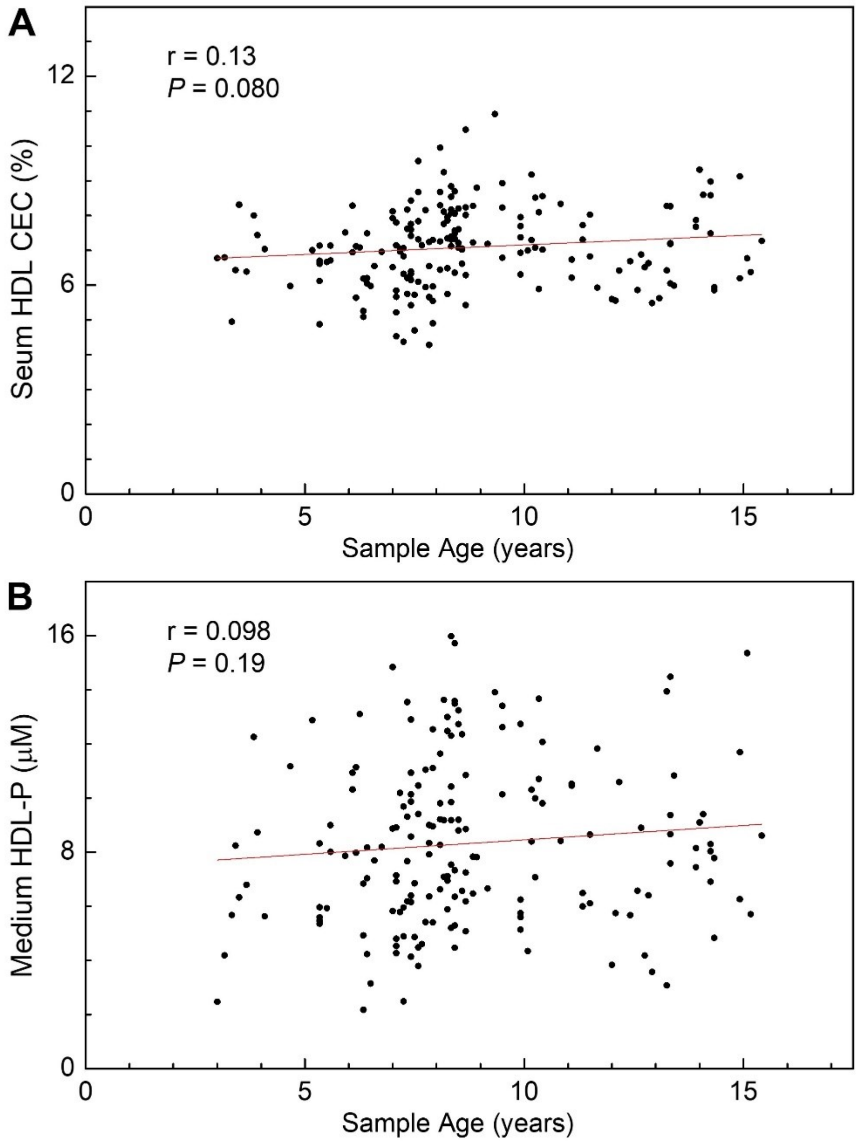

Impact of plasma storage time without cryoprotectants on (A) the cholesterol efflux capacity or (B) the concentration of medium sized HDL particles of freshly isolated serum HDL. Plasma samples from 179 subjects were collected over more than 15 years without including any cryoprotectants for sample storage. The 179 subjects were a subset of subjects randomly selected from participants with or without severe carotid cerebrovascular disease (CCVD) and with or without diabetes enrolled in the CLEAR study (1) for sample stability analysis upon extended storage time. Serum HDL was freshly isolated for measurement of cholesterol efflux capacity (CEC) using cAMP stimulated J774 macrophages and concentrations of different sized HDL particles by calibrated ion mobility analysis (cIMA).

1. Jarvik, G. P., L. S. Rozek, V. H. Brophy, T. S. Hatsukami, R. J. Richter, G. D. Schellenberg, and C. E. Furlong. 2000. Paraoxonase (PON1) phenotype is a better predictor of vascular disease than is PON1(192) or PON1(55) genotype. *Arterioscler Thromb Vasc Biol* **20**: 2441-2447.

Supplemental Table 1. HDL particle concentrations and sizes of the CPROBE subjects

HDL Particles	Control	CVD	P-value
Number of subjects	46	46	
Total HDL Particle (µM)	20.26 (17.82-24.08)	19.65 (14.66-21.83)	0.11*
Extra small HDL Particle (µM)	1.27 (0.92-1.46)	1.24 (0.99-1.68)	0.64†
Small HDL Particle (µM)	5.38 (4.07-7.26)	5.37 (4.40-7.27)	0.53†
Medium HDL Particle (µM)	6.62 (5.34-8.63)	5.65 (4.14-6.90)	0.015*
Medium large HDL Particle (µM)	1.98 (0.93-2.68)	1.67 (0.81-2.46)	0.61†
Large HDL Particle (µM)	3.82 (2.43-5.74)	3.06 (2.11-4.57)	0.13†
Extra-large HDL Particle (µM)	0.81 (0.47-1.40)	0.77 (0.38-1.31)	0.62†
Extra small HDL size (nm)	7.59 (7.56-7.62)	7.58 (7.52-7.60)	0.10†
Small HDL size (nm)	8.26 (8.23-8.31)	8.24 (8.20-8.31)	0.13†
Medium HDL size (nm)	9.08 (9.02-9.11)	9.04 (8.98-9.10)	0.080†
Medium large HDL size (nm)	9.70 (9.63-9.81)	9.68 (9.61-9.74)	0.13*
Large HDL size (nm)	10.89 (10.77-10.98)	10.85 (10.60-10.95)	0.15†
Extra-large HDL size (nm)	12.29 (12.20-12.45)	12.27 (12.20-12.39)	0.56†

Entries are medium (IQR). *P*-values are from a Student's *t*-test for normally distributed variables (*) or a Mann-Whitney U test for abnormally distributed variables (†). Shapiro-Wilk test was used for the normality test.

Supplemental Table 2. Odds Ratio of medium HDL-P for incident CVD after adjusting for potential confounders in the CPROBE cohort

	Model 1		Model 2		Model 3		Model 4	
**Covariate**	**OR (95% CI)**	**p-value**	**OR (95% CI)**	**p-value**	**OR (95% CI)**	**p-value**	**OR (95% CI)**	**p-value**
Medium HDL-P	0.57 (0.36-0.92)	0.022	0.53 (0.32-0.89)	0.015	0.55 (0.32-0.97)	0.039	0.45 (0.22-0.93)	0.032
Age	1.06 (0.67-1.68)	0.79	1.03 (0.62-1.70)	0.92	1.19 (0.70-2.04)	0.52	2.03 (0.94-4.39)	0.071
HTN	1.08 (0.41-2.90)	0.87	0.83 (0.28-2.46)	0.73	0.66 (0.21-2.07)	0.48	1.12 (0.27-4.69)	0.88
Present Smoker			1.08 (0.29-4.06)	0.91	1.28 (0.33-5.06)	0.72	2.33 (0.30-17.8)	0.42
BMI			1.29 (0.80-2.10)	0.29	1.23 (0.75-2.03)	0.41	1.26 (0.60-2.63)	0.54
eGFR			0.85 (0.52-1.39)	0.51	0.82 (0.49-1.38)	0.46	1.14 (0.57-2.25)	0.71
LDL-C					1.78 (0.98-3.22)	0.059	0.79 (0.38-1.66)	0.54
Log(TGs)					1.25 (0.78-2.00)	0.35	1.16 (0.53-2.52)	0.71
HDL-C					0.78 (0.44-1.39)	0.40	0.78 (0.40-1.52)	0.47
Log(CRP)							1.93 (0.97-3.85)	0.060
UPC Ratio							3.08 (1.43-6.61)	0.0040

Adjusted odds ratios and p-values were obtained from multivariate logistic regression analysis. The levels of medium HDL-P and clinical confounders and lipid risk factors were used as independent variables and incident CVD was the outcome in the logistic regression analysis. Odds ratios are per SD increase of medium HDL-P. Model 1 is a model adjusted for age and hypertension (diabetic status and sex were matched). Model 2 is model 1 further adjusted for other potential clinical confounders including present smoker, BMI, and eGFR. Model 3 is

model 2 further adjusted for lipid risk factors including LDL-C, log transformed TGs, and HDL-C. Model 4 is model 3 further adjusted for CRP and urinary protein to creatinine (UPC) ratio.

Supplemental Table 3. HDL particle concentrations and sizes of the CRIC cohort

HDL Particles	Control	CVD	P-value
Number of subjects	57	34	
Total HDL Particle (µM)	12.27 (9.85-14.25)	9.60 (8.54-13.14)	0.0073
Extra small HDL Particle (µM)	0.74 (0.58-0.98)	0.75 (0.51-1.00)	0.55
Small HDL Particle (µM)	3.28 (2.55-3.92)	2.99 (2.12-3.88)	0.29
Medium HDL Particle (µM)	4.51 (3.74-5.69)	3.62 (3.03-4.59)	0.0032
Medium large HDL Particle (µM)	0.58 (0.37-0.84)	0.51 (0.35-0.72)	0.24
Large HDL Particle (µM)	1.66 (1.02-2.67)	1.19 (0.75-2.46)	0.12
Extra-large HDL Particle (µM)	0.65 (0.36-1.04)	0.46 (0.27-1.00)	0.16
Extra small HDL size (nm)	7.52 (7.50-7.55)	7.51 (7.48-7.54)	0.28
Small HDL size (nm)	8.17 (8.14-8.23)	8.15 (8.13-8.19)	0.12
Medium HDL size (nm)	9.01 (8.97-9.08)	8.98 (8.96-9.08)	0.25
Medium large HDL size (nm)	9.84 (9.80-9.89)	9.85 (9.81-9.90)	0.54
Large HDL size (nm)	10.79 (10.74-10.86)	10.80 (10.73-10.90)	0.36*
Extra-large HDL size (nm)	12.12 (12.01-12.21)	12.13 (11.97-12.28)	0.93

Entries are medium (IQR). *P*-values are from a Student's *t*-test for normally distributed variables (*) or a Mann-Whitney U test for abnormally distributed variables. Shapiro-Wilk test was used for the normality test.

Supplemental Table 4. Odds Ratio of medium HDL-P for incident CVD after adjusting for potential confounders in the CRIC cohort

	Model 1		Model 2		Model 3		Model 4	
**Parameter**	**OR (95% CI)**	**p-value**	**OR (95% CI)**	**p-value**	**OR (95% CI)**	**p-value**	**OR (95% CI)**	**p-value**
mHDL-P	0.51 (0.29-0.89)	0.018	0.47 (0.25-0.86)	0.015	0.38 (0.18-0.77)	0.0079	0.42 (0.20-0.87)	0.019
Age	0.96 (0.92-1.01)	0.12	0.95 (0.91-1.01)	0.079	0.95 (0.90-1.01)	0.075	0.71 (0.40-1.25)	0.23
Hypertension	2.47 (0.47-13.12)	0.29	2.62 (0.48-14.19)	0.26	2.64 (0.46-15.14)	0.28	2.08 (0.36-11.9)	0.41
Female	0.77 (0.30-2.00)	0.59	0.93 (0.33-2.62)	0.89	0.47 (0.12-1.81)	0.27	0.40 (0.097-1.64)	0.20
Diabetes	0.96 (0.37-2.46)	0.92	1.07 (0.41-2.82)	0.88	1.25 (0.45-3.49)	0.67	1.18 (0.41-3.41)	0.76
Present smoker			0.87 (0.23-3.22)	0.83	1.21 (0.31-4.83)	0.78	1.54 (0.37-6.41)	0.55
BMI			0.96 (0.90-1.02)	0.21	0.98 (0.92-1.04)	0.44	0.80 (0.46-1.40)	0.44
eGFR			1.01 (0.96-1.05)	0.80	1.00 (0.96-1.06)	0.86	1.18 (0.66-2.14)	0.58
HDL-C					1.06 (1.00-1.11)	0.038	2.07 (0.96-4.46)	0.062
LDL-C					1.00 (0.99-1.01)	0.90	0.92 (0.54-1.57)	0.76
Log(TGs)					13.74 (1.28-147)	0.030	1.57 (0.89-2.78)	0.12
UPC Ratio							2.16 (0.80-5.84)	0.13

Adjusted odds ratios and p-values were obtained from multivariate logistic regression analysis. The levels of medium HDL-P and clinical confounders and lipid risk factors were used as independent variables and incident CVD was the outcome in the logistic regression analysis. Odds ratios are per SD increase of medium HDL-P and other continuous variables. Model 1 is a model adjusted for age, hypertension, sex, and diabetes.

Model 2 is model 1 further adjusted for other potential clinical confounders including present smoker, BMI, and eGFR. Model 3 is model 2 further adjusted for lipid risk factors including LDL-C, log transformed TGs, and HDL-C. Model 4 is model 3 further adjusted for urinary protein to creatinine ratio (UPC ratio).
